# Bile Duct Injuries after Cholecystectomy: An Individual Patient Data Systematic Review

**DOI:** 10.3390/jcm13164837

**Published:** 2024-08-16

**Authors:** Paolo Vincenzi, Federico Mocchegiani, Daniele Nicolini, Andrea Benedetti Cacciaguerra, Diletta Gaudenzi, Marco Vivarelli

**Affiliations:** 1 Division of HPB and Abdominal Transplant Surgery, Department of Gastroenterology and Transplants, Azienda Ospedaliero-Universitaria delle Marche, 60126 Ancona, Italy; paolo.vincenzi@ospedaliriuniti.marche.it (P.V.); daniele.nicolini@ospedaliriuniti.marche.it (D.N.); diletta.gaudenzi@ospedaliriuniti.marche.it (D.G.); 2 Division of HPB and Abdominal Transplant Surgery, Department of Experimental and Clinical Medicine, Polytechnic University of Marche, 60126 Ancona, Italy; andrea.benedetticacciaguerra@ospedaliriuniti.marche.it (A.B.C.); vivarelli63@libero.it (M.V.)

**Keywords:** cholecystectomy, bile duct injuries, treatment failure, risk factors, hepaticojejunostomy, end-to-end anastomosis, endoscopic retrograde cholangiopancreatography, percutaneous transhepatic biliary drainage

## Abstract

**Background**: Post-cholecystectomy bile duct injuries (BDIs) represent a challenging complication, with negative impacts on clinical outcomes. Several surgical and endoscopic/interventional radiologist (IR) approaches have been proposed to manage these damages, though with high failure rates. This individual patient data (IPD) systematic review analyzes the potential risk factors for failure after treatment interventions for BDIs, both surgical and endoscopic/IR. **Methods**: An extensive literature search was conducted on MEDLINE and Scopus for relevant articles published in English on the management of BDIs after cholecystectomy, between 1 January 2010 and 31 December 2023. Our series of BDIs was included. BDIs were always categorized according to the Strasberg’s classification. The composite primary endpoints evaluated were the failure of treatment interventions, defined as patient death or the requirement of any other procedure, whatever surgical and/or endoscopic/IR, after the primary treatment. **Results**: A total of 342 cases were retrieved from our literature analysis, including our series of 19 patients. Among these, three groups were identified: “upfront surgery”, “upfront endoscopy and/or IR” and “no upfront treatment”, consisting of 224, 109 and 9 patients, respectively. After eliminating the third group, treatment intervention failure was observed overall in 34.2% (114/333) of patients, of whom 80.7% (92/114) and 19.3% (22/114) in the “upfront surgery” and in the “upfront endoscopy/IR” groups, respectively. At multivariable analysis, injury type D and E, and repair in a non-specialized center represented independent predictors of treatment failure in both groups, whereas laparoscopic cholecystectomy (LC) converted to open and immediate attempt of surgical repair exclusively in the first group. **Conclusions**: Significant treatment failure rates are responsible for remarkable negative effects on immediate and longer-term clinical outcomes of post-cholecystectomy BDIs. Understanding the important risk factors for this outcome may better guide the most appropriate therapeutical approach and improve clinical decisions in case this serious complication occurs.

## 1. Introduction

Since the first description in 1985 [1], laparoscopic cholecystectomy (LC) has gradually emerged as the gold standard surgical technique in the treatment of gallstone disease and acute acalculous cholecystitis, representing one of the most common procedures performed worldwide in both elective and urgent settings [2,3].

Though with improving surgical experience and technological advances, bile duct injuries (BDIs) might still occur at a frequency ranging between 0.1 and 5.2%, with the highest percentages reported in the context of acute cholecystitis [2,3,4,5], compared to the 0.2–0.3% rate historically documented in open cholecystectomy (OC) [6]. 

BDIs are associated with significant morbidity, mortality, and considerable costs for the healthcare system [2,3,4,5,7], meaning at the same time a very common cause of litigation against general surgeons [8].

Several classifications of BDIs have been proposed by different authors [9,10,11,12], with Strasberg’s [10] currently representing the most used in clinical practice [13], allowing a simple and quick differentiation between minor and major BDIs [4].

Nevertheless, since vascular injuries might be found in 10–60% of iatrogenic BDIs [14,15], the more recent ATOM (anatomic, time of detection, mechanism) classification, proposed by the European Association for Endoscopic Surgery (EAES) in 2013 [16], integrates the main previously described codification systems into a composite, all-inclusive, nominal scheme combining bile tract anatomical damage, vascular injury, timing of detection, and mechanism of damage, with the purpose of standardizing BDI definitions, though limited by a poor clinical application due to its complexity [4].

Previous systematic reviews have focused their attention on the analysis of large-scale nationally validated databases, population-based cohort studies [5,17], and pooled data extracted through selectively screening major medical databases [18,19,20], with the associated weaknesses mainly consisting of limited accuracy, reporting bias and several confounders. 

In order to overcome these limitations, we decided to conduct an individual patient data (IPD) systematic review as an alternative to a conventional systematic review, based on the search of all available case reports and series describing BDIs after cholecystectomy, aiming at providing a valid estimate of the main features inherent this subject, the treatment effects and their outcomes. In addition, the predictors of outcome when a surgical and/or an endoscopic/interventional radiology (IR) approach was adopted, were evaluated.

## 2. Materials and Methods

### 2.1. Data Sources and Extraction

An extensive literature search was conducted on MEDLINE and Scopus for published relevant articles. MeSH terms for the search string are provided in Appendix A. The search was limited to papers published in English between 1 January 2010 and 31 December 2023.

The case series of BDIs managed at our institution during the study period was also included.

The abstracts of the citations identified by the database search were independently screened by two authors (P.V. and A.B.C.). When it was certain from the abstract that the article was not of use, it was excluded. From all other studies, we obtained the full article to decide whether the study was potentially eligible. Discrepancies were resolved by discussion. 

In addition, reference lists in all extracted studies were manually searched for eligible citations. 

All published clinical studies that included the management of patients with BDIs after cholecystectomy were selected for further analysis. The exclusion criteria were as follows: (1) cholecystectomy not indicated for acute or chronic onset of cholelithiasis and for acute acalculous cholecystitis; (2) reviews, including those with pooled data, and editorials; (3) studies not precisely describing the type of BDI; (4) studies reporting a BDI not related to a procedure of cholecystectomy; (5) studies not describing the diagnostic or treatment modalities employed; (6) studies without sufficient data for analysis. 

Duplicate studies were also identified and excluded. 

Patients with multiple concomitant injuries to the biliary tree and/or vascular injuries (VIs) were included in the analysis, and exclusively the most serious damage to the biliary tree was recorded.

BDIs were always categorized according to the Strasberg classification [10]. In case a different or any classification was used in the paper included in the analysis, all the information provided were accurately reviewed in order to sort the BDI by the Strasberg codification [10].

The authors of the included studies were contacted only when the IPD needed for the analysis were incompletely described in the manuscript. 

The available published studies regarding BDIs after cholecystectomy consist of case reports or single-center case series (Table A1, Appendix A). The choice of management of these injuries, whether surgical, endoscopic, IR, or conservative, was based on local expert opinion.

**Figure 1:**
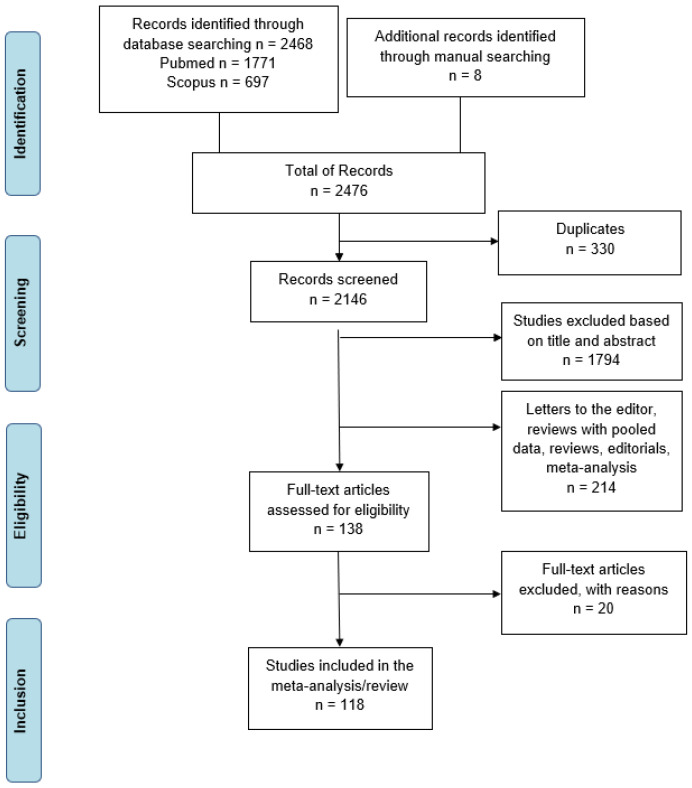
Literature search PRISMA flow diagram.

The PRISMA (preferred reporting items for systematic reviews and Meta-analyses) checklist was used to perform this systematic review [21] (Figure 1).

The methodological quality and the risk of bias of the included studies were assessed by two independent reviewers (P.V. and A.B.C.) through the JBI critical appraisal tools for case series and case reports studies [22].

This review was registered as PROSPERO CRD42024551515.

### 2.2. Study Details and Endpoints

The following details were recorded for each study: year of publication, authors, title, journal, design (case report or case series), and number of cases. The study population demographics as well as clinical parameters, such as type of cholecystectomy (open or laparoscopic), conversion to open during LC and causes, the interval between BDI occurrence and recognition (timing of diagnosis), type of surgical treatment applied, timing of surgical repair, type of endoscopic, and IR treatment employed and referral to an hepato-pancreato-biliary (HPB) or tertiary care center, were also extracted. Mortality directly correlated to the event analyzed and long-term outcomes were also documented. 

Some baseline variables, such as emergent and elective cholecystectomy, were not analyzed due to the high percentage of missing data.

All the baseline and outcome variables analyzed are listed in Table 1. 

Three main groups were identified: group one, named “upfront surgery”, was composed of all patients undergoing a primary attempt of surgical repair of the BDI while in group two, named “upfront endoscopy and/or IR”, all cases were included that were initially treated by endoscopic or IR methods. The third group was defined “no upfront treatment”, since neither a surgical or an endoscopic/IR approach was undertaken, and a conservative management was pursued.

The techniques included in the first two groups were all those meant to provide an effective management of the BDI and therefore represented by suture or clips placement, T-tube insertion, end-to-end duct-to-duct anastomosis, hepatico-jejunostomy (HJ), hepatic resection (HR) in the group “upfront surgery” and by the placement of endoprosthesis or naso-biliary (NB) drainage through endoscopic retrograde colangiopancreatography (ERCP), by percutaneous transhepatic biliary drainage (PTBD) or other IR methodic, such as embolization coils, sclerotherapy and fibrin glue, in the other group.

Timing of BDI diagnosis was divided in two intervals: within one week and after one week the initial cholecystectomy. 

Timing of surgical repair of BDI was defined as immediate if occurring within one day from the cholecystectomy, early if after one day and within one week, delayed if after one week and within 6 weeks and late if after 6 weeks [23,24]. 

The composite primary endpoints evaluated were the failure of treatment interventions, defined as patient death or the requirement of any other procedure, whatever surgical and/or endoscopic/IR, after the primary treatment. 

### 2.3. Statistical Analysis

IPD extracted from eligible studies were entered in an Excel spreadsheet (Microsoft, Redmond, WA, USA).

Frequency distributions were determined for baseline categorical variables, and the arithmetic mean along with standard error (±SE) was calculated for baseline continuous variables [with median and corresponding interquartile (IQ) range being used for baseline continuous variables having skewed distributions]. 

Tests of association between baseline variables and treatment intervention failure development (No: no failure/Yes: failure) were performed using Pearson (uncorrected) chi-squared tests for dichotomous baseline variables and standard *t*-tests or Mann–Whitney tests as appropriate for continuous baseline variables (using natural logarithmic transformed values for skewed distributions). 

Multivariable analysis was performed using stepwise logistic and linear regression on those variables with statistical relevance at the univariable analysis (*p*-value < 0.05).

The optimal cut-off for continuous variables was obtained from analyses of receiver operating characteristic (ROC) curves.

The statistical analysis was performed using IBM SPSS Statistics for Windows, version 24 (IBM Corp., Armonk, NY, USA).

## 3. Results

### 3.1. Study Population

The above-mentioned key words yielded a total of 323 cases from 118 manuscripts [25,26,27,28,29,30,31,32,33,34,35,36,37,38,39,40,41,42,43,44,45,46,47,48,49,50,51,52,53,54,55,56,57,58,59,60,61,62,63,64,65,66,67,68,69,70,71,72,73,74,75,76,77,78,79,80,81,82,83,84,85,86,87,88,89,90,91,92,93,94,95,96,97,98,99,100,101,102,103,104,105,106,107,108,109,110,111,112,113,114,115,116,117,118,119,120,121,122,123,124,125,126,127,128,129,130,131,132,133,134,135,136,137,138,139,140,141,142], as displayed in Table A1 (Appendix A). Most of the case studies (*n* = 88, 74.6%) were isolated case reports, with the remaining 30 (25.4%) reporting between 2 and 43 cases of BDI.

Adding our case series composed of 19 cases (Table A2, Appendix A), the entire population analyzed in the review was made of 342 cases.

In more than half of the studies (67/118, 56.7%), the authors did not utilize any classification system to report the BDI, whereas in 28.8% (*n* = 34) of the studies identified, the Strasberg’s classification was used to catalogue the injury type [10]. The Bismuth–Corlette [9], Stewart–Way [11], ATOM [16], Hannover [12], Neuhaus [143], and Bergman [144] codification systems were applied in the remaining studies (Table A1, Appendix A). 

Among the cases identified, the group “upfront surgery” consisted of 224 patients (65.5%), whereas the groups “upfront endoscopy and/or IR” and “no upfront treatment” of 109 (31.9%) and 9 (2.6%) cases, respectively.

Due to the scarce number of patients belonging to the “no upfront treatment” group and since this topic was not incorporated among our primary endpoints, it was excluded from further analysis. 

At the comparative analysis, the overall rate of LC: converted to open and, specifically, the conversion rate after recognizing a BDI, the degree of bile duct damage, according to the Strasberg’s classification [10], and the percentage of associated VIs differed between the two groups analyzed, with significantly higher percentages of converted procedures, and of complex injuries, i.e., types D and E, with concomitant vascular damage in the “upfront surgery” group (*p* = 0.001 and <0.0001, respectively), as outlined in Table 2.

Likewise, among the other baseline variables investigated, a significantly higher rate of early diagnosis, i.e., within one week, and of surgical repair in a non-specialized center, were observed in the same group (*p* < 0.0001 and 0.025, respectively) (Table 2).

Accordingly, when comparing the primary endpoint, the occurrence of treatment intervention failure was significantly worse in the surgical group compared to the endoscopy/IR group (*p* < 0.0001) and consequently the mortality rate (*p* = 0.025), as shown in Table 2.

### 3.2. Upfront Surgery Group

#### 3.2.1. Distributions of Baseline Characteristics

Overall, median patient age was 47 (IQ range: 36.2–62) years, with 13% of patients (24/184) aged more than 70 years old and 37.1% (75/202) of male sex, as shown in Table 2. Indeed, age and sex were not reported in 40 and 22 cases, respectively.

Cholecystectomy was performed laparoscopically in the majority of cases (*n* = 197, 87.9%), and converted to open in approximately one quarter of cases (*n* = 55, 27.9%), mainly because of intraoperative recognition of a BDI, as shown in Table 2.

Type E (*n* = 152, 67.9%) and D (*n* = 44, 19.6%) lesions, according to the Strasberg’s classification [10], represented the most frequent indications for surgery, followed by type C (*n* = 13, 5.8%), A (*n* = 11, 4.9%), and B (*n* = 4, 1.8%) injuries, as displayed in Table 2.

Of note, in the type E category of BDIs, the E2 and E4 subtypes were the most common damage patterns, reported in 58 (38.2%) and 40 (26.3%) cases, respectively, followed by the E3 (26/152, 17.1%) and the E1 (23/152, 15.1%) patterns. The most serious BDI, i.e., the E5 subtype, was recorded in only five cases (3.3%).

An associated VI was documented in almost one quarter of patients (51/224, 22.8%) (Table 2), with the RHA involved in 82.3% (*n* = 42) of cases and the proper (PHA) or common hepatic artery (CHA) in approximately 10% of cases (*n* = 5). A combination of hepatic artery (HA) and PV damage was reported in 11 patients (21.6%).

Of the 190 patients (84.8%) with mentioned timing of BDI diagnosis, in the majority (*n* = 151, 79.5%), the BDI was recognized within one week from the cholecystectomy (Table 2).

HJ was the most common surgical technique adopted for the repair of BDIs, utilized in half of cases (113/224, 50.4%), followed by leak repair through application of stitches or clips and end-to-end duct-to-duct anastomosis, chosen in 43 (19.2%) and 25 (11.2%) cases, respectively.

HR, generally consisting of right hepatectomy or right posterior sectorectomy, alone or in association with HJ, comprised the repair methods applied in 3.6% (*n* = 8) and 5.4% (*n* = 12) of cases, respectively, whereas in 4.5% of patients (*n* = 10), stitches or clips removal at the level of the common bile duct (CBD), omental patch or hepatico-duodenostomy, were the other methodic employed to fix the damage. 

Lastly, a T-tube placement, alone or combined with other procedures, was carried out in 38 patients (17%).

Of the 204 (91%) patients with reported timing of surgical repair, an immediate repair was performed in 44.1% (*n* = 90) of cases, whereas an early, delayed, and late approach of reconstruction was chosen in 39 (19.1%), 43 (21.1%) and 32 (15.7%) cases, respectively. 

In the majority of patients (79%, 177/224), the BDI was repaired in a specialized HPB center or in a tertiary care facility, whereas in the remaining cases (21%, 47/224), the surgical repair was attempted at the community hospital where the procedure of cholecystectomy was performed, as outlined in Table 2.

#### 3.2.2. Distribution of the Primary Endpoint

In total, 92 patients (41%) developed a failure of the initial surgical treatment intervention (Table 2).

The most frequent reason for failure was the development of a secondary stricture at the level of the CBD or of the previous anastomosis (*n* = 53, 57.6%), followed by a persistent bile leak (*n* = 23, 25%) and the occurrence of a secondary biliary cirrhosis (SBC) over time (*n* = 7, 7.6%).

Perioperative mortality was reported in 10 patients (10.9%) (Table 2).

In the 82 alive patients who required a secondary intervention, HJ was carried out in 57.3% (*n* = 47) of cases, whereas an isolated HR and IR/endoscopic procedures were completed in approximately 33% (*n* = 27) and 42% (*n* = 34) of cases, respectively.

In 22 patients (26.8%), HJ was combined with HR, whereas 7 patients (8.5%) underwent a liver transplantation as a result of SBC.

#### 3.2.3. Univariable Comparisons of Baseline Variables between Failure and Non-Failure of the Surgical Treatment Intervention

Tests of the association of baseline variables with development of surgical treatment intervention failure found that patients undergoing upfront OC, LC converted to open for any reason, experiencing a major BDI, i.e., type D and E, and hence sustaining an immediate repair, i.e., within one day from cholecystectomy, were more likely to develop this outcome (*p* = 0.024, 0.001, 0.014, and 0.001, respectively) (Table 3). 

Among the other variables analyzed, a repair of the injury in an HPB/tertiary care institution was associated with a significantly lower percentage of failure (31.6%, 56/177) compared to a repair attempted in a non-specialized center (76.6%, 36/47) [*p* < 0.0001], as displayed in Table 3.

#### 3.2.4. Multivariable Analysis Results

In a stepwise logistic regression analysis of the baseline predictors of surgical failure, three variables were selected containing independent predictive value: injury type D and E (*p* = 0.0094), immediate attempt of surgical repair (*p* = 0.0113), and repair in a non-specialized center (*p* = 0.001) (Table 4). 

Once these three multivariable predictors of surgical treatment intervention failure were controlled, none of the other baseline variables offered additional predictive value. 

Of note, three of the baseline variables were highly correlated and collinear: LC converted to open, and LC converted to open because of BDI recognition and immediate attempt of surgical repair (*p* = 0.001). Thus, it was statistically impossible to determine which of these three baseline variables were the most appropriate predictor of surgical failure development.

However, it appeared that clinically, the immediate attempt of surgical repair was the most appropriate variable to be selected into the surgical failure logistic model, as the timing of surgical repair clearly provides the most accurate information on the management of BDIs. 

### 3.3. Upfront Endoscopy/IR Group

#### 3.3.1. Distributions of Baseline Characteristics

Overall, median patient age was 52 (IQ range: 42–65) years, with 15.8% of patients (16/101) aged more than 70 years old and 42.2% (43/102) of male sex, as shown in Table 2. Indeed, age and sex were not reported in eight and seven cases, respectively.

Cholecystectomy was performed laparoscopically in the majority of cases (*n* = 96, 88%) and converted to open in only ten percent of cases (10/96), as shown in Table 2.

The most frequent indications for an endoscopic and/or IR treatment were represented by type E (*n* = 50, 45.9%) and A (*n* = 30, 27.5%) lesions, followed by type C (*n* = 18, 16.5%), D (*n* = 10, 9.2%) and B (*n* = 1, 0.9%) injuries, as outlined in Table 2.

Of note, in the type E category of BDIs, the E2 and E1 subtypes were the most common damage patterns, reported in approximately one third (*n* = 18, 36%) and one quarter (*n* = 12, 24%) of cases, respectively, followed by the E4 (10/50, 20%) and the E3 (7/50, 14%) patterns. The most serious BDI, i.e., the E5 subtype, was recorded in only three cases (6%).

Similarly, an associated VI was documented in only three patients (3/109, 2.8%), with the exclusive involvement of the RHA (Table 2).

Of the 101 patients (92.7%) with the mentioned timing of BDI diagnosis, in more than half (*n* = 57, 56.4%), the BDI was recognized at least one week after the initial operation of cholecystectomy (Table 2).

Among the endoscopic procedures chosen for the management of BDIs, ERCP with stenting and/or NB drainage was the most common technique, adopted in almost all cases (92/93, 98.9%).

IR methods were employed in almost half of the patients (53/109, 48.6%), mainly consisting of PTBD, utilized in approximately three quarter of cases (39/53, 73.6%), followed by leak repair through coil embolization, sclerotherapy with acetic acid or ethanol, and fibrin glue application, employed in 9 (17%), 2 (3.8%), and 1 (1.8%) case. 

Combined ERCP and IR, but mainly PTBD, were delivered in approximately one third of cases (38/109, 34.9%), whereas a real rendez-vous procedure was described in almost one quarter of patients (24/109, 22%).

In the majority of patients (97/109, 89%), the BDI was managed in a specialized HPB center or in a tertiary care facility, whereas in the remaining cases (12/109, 11%), the endoscopic/IR procedure was carried out at the community hospital where cholecystectomy was performed (Table 2).

#### 3.3.2. Distribution of the Primary Endpoint

In total, 22 patients (20.2%) developed a failure of the initial endoscopic/IR treatment intervention (Table 2).

The most frequent reason for failure was the persistence of the bile leak (*n* = 15, 68.2%), followed by a secondary stricture at the level of the CBD (*n* = 7, 31.8%).

Perioperative mortality was not reported in this group (Table 2).

In the 22 patients who required a secondary intervention, HJ was carried out in the majority of cases (*n* = 18, 81.8%), whereas an isolated HR and a combined procedure of HJ and HR were completed in approximately half (*n* = 10, 45.5%) and one third of cases (*n* = 7, 31.8%). In the only remaining case (4.5%), another ERCP with stent placement was performed in order to treat the CBD secondary stricture. 

#### 3.3.3. Univariable Comparisons of Baseline Variables between Failure and Non-Failure of the Endoscopic/IR Treatment Intervention

Tests of association of baseline variables with the development of endoscopic/IR treatment intervention failure found that patients presenting with a higher degree of BDIs, i.e., type D and E, and with an associated VI, were more likely to develop this outcome (*p* = 0.001 and 0.006, respectively) (Table 5). 

Among the other variables analyzed, the management of the injury in an HPB/tertiary care institution was associated with a significantly lower percentage of failure (13.4%, 13/97) compared to that when attempted in a non-specialized center (75%, 9/12) [*p* < 0.0001], as displayed in Table 5.

#### 3.3.4. Multivariable Analysis Results

In a stepwise logistic regression analysis of baseline predictors of endoscopic/IR failure, two variables were selected containing independent predictive value: injury type D and E (*p* = 0.02), and repair in a non-specialized center (*p* = 0.0002) (Table 6). 

Once these two multivariable predictors of endoscopic/IR treatment intervention failure were controlled, none of the other baseline variables offered additional predictive value. 

### 3.4. Sensitivity Analysis

The sensitivity analysis was performed by including only the most recent studies, published between 2013 and 2023.

In the “upfront surgery group”, this sensitivity analysis confirmed the predictive factors emerged at the overall analysis, whereas in the “upfront endoscopy/IR group”, only the favorable effect of repair in an HPB/tertiary care center was still preserved (Table A3, Appendix A).

## 4. Discussion

Our study represents the first IPD review and one of the largest case series analyzing the current management of BDIs after cholecystectomy, with a particular focus on investigating the baseline predictors of treatment failure, both surgical and endoscopic/IR.

Among all the baseline variables assessed, we were able to identify specific risk factors for developing this outcome in those patients presenting with a biliary tract damage after cholecystectomy (LC converted to open for any reason, severity of BDI, immediate surgical reconstruction, and repair in a non-specialized center). 

Overall, in our study population, significantly higher incidences of treatment failure and mortality were reported in the “upfront surgery” group when compared to the “upfront endoscopy/IR” group. However, this finding should not lead one to conclude that an upfront surgical approach in the case of BDI after cholecystectomy might actually increase the failure risk. In fact, higher rates of conversion to open, mainly because of BDI recognition, major degrees of damage patterns with concomitant vascular impairment, the early diagnosis of the injury and hence the immediate attempt of repair in a non-specialized center, emerged as significant baseline variables in the surgical group, suggesting that more complex injuries requiring conversion to open were recognized earlier and thus potentially repaired in the non-specialized hospital where the cholecystectomy was performed, confirming the literature findings [4,145]. 

Thus, the prognostic value of a surgical approach in terms of it implying a greater risk of failure appears to be more of a reflection of the patient’s clinical status at the time of the damage rather than a direct consequence of the surgical treatment itself.

When analyzing both groups separately, “upfront surgery” and “upfront endoscopy/IR”, the severity of BDI emerged as a significant risk factor for treatment failure.

Indeed, from what emerged in our IPD review, though the majority of the series included did not use any classification system to categorize the reported BDIs and though there is still no consensus on the “gold standard” classification for these injuries, we decided to adopt Strasberg’s classification [10], principally due to its reliable application in clinical practice, allowing to easily convert the clinical information detailed in each case report or series to the accurate grade of damage. 

Conversely, more recent and extremely detailed codifications systems, such as the ATOM [16], properly developed to standardize the definition of BDI and improve the reproducibility of data in epidemiological and comparative studies, might lack applicability in the current clinical scenario due to its complexity [4], as emerged in the analyzed reports.

In our study, indeed, grade D and E carried an increased risk of treatment failure by approximately four times in both the groups investigated. While in the surgical group, these types of injuries represented the most common damage patterns, accounting alone for almost 90% of cases, in the other group, approximately half of patients presented with these degrees of damage.

In the original classification by Strasberg et al. [10], a type D injury was defined as a lateral injury to the major bile duct whereas a type E injury consisted of a stricture affecting the CBD and/or the confluence of lobar ducts and was further divided into five subtypes of increasing severity, directly correlated to the involvement of the right and left hepatic ducts. 

Unfortunately, a wide range of injuries included within the D category, ranging from a small clean lateral laceration to a laceration greater than 50% of circumference or a thermal injury whose full extent may be difficult to determine initially, explains the reasons underlying an objective difficulty in classifying the severity of this damage, leading to concomitant controversies in their management [13]. 

The other three categories of BDIs in the Strasberg’s classification are represented by type A, B and C, the first one described as a leak from the cystic duct and/or the duct of Luschka while the second and the third as an occlusion and a transection, without ligation, respectively, of an aberrant right hepatic duct [10]. 

In our study, type A injury represented the second most common damage pattern in the “endoscopy/IR” group, documented in one quarter of cases, as a result of its approachability with ERCP, followed by the type C, whereas the grade B damage pattern was uncommon in both groups.

Since the last two refer to aberrant right hepatic ducts, it is often difficult to reach a correct diagnosis, and consequently, their management might be complex, independently from adopting an endoscopic or surgical treatment [4].

As stated above, Strasberg’s classification [10] did not mention an associated VI, which was very frequent in the surgical group, mainly involving the arterial side, though combined lesions of the HA and PV were described in one quarter of cases, and conversely quite rare in the other group analyzed. However, the concomitant presence of vascular damage did not interfere with the response to treatment. 

Regarding the appropriate timing of surgical repair, it has been a common matter of debate in the literature, with two metanalyses demonstrating a significantly lower risk of treatment failure, post-repair bile leak, need for surgical revision, and overall morbidity when a delayed strategy, i.e., repair after 6 weeks from the cholecystectomy, was adopted, compared to an early approach, i.e., within 6 weeks [145,146].

In addition, on-table repair, by direct suture or bilio-enteric anastomosis and mainly if carried out by non-HPB specialists, presented a trend for a higher risk of failure in comparison to postoperative repair [145]. 

On the other hand, recent evidence from a large multicenter study conducted by the European-African Hepato-Pancreato-Biliary Association (E-AHPBA) [24], together with a randomized controlled trial [147], indicates that the timing of biliary reconstruction with HJ after major post-cholecystectomy BDIs seems not to have any impact on the analyzed outcomes: a successful reconstruction rate, anastomotic patency, re-intervention rate, morbidity, and mortality.

In a cost-effective analysis on more than 500,000 cholecystectomies, Sweigert et al. [148] demonstrated that a delayed repair strategy, i.e., after 6 weeks, though not associated with increased mortality, was responsible for significantly higher inpatient costs (+USD 45,111; 95% CI: USD 36,813–USD 53,409) compared to an early approach, defined in this study as within 3 days from the cholecystectomy, whereas significantly higher costs and mortality were reported when an intermediate repair, from 4 days to 6 weeks, was chosen, thus suggesting that the latter option be avoided.

However, due to the lack of a standardized definition for the timing of BDI repair, a recent systematic review stated that no definitive conclusions can be drawn on the optimal interval period to fix these damages, at the same time advocating for a uniform reporting system [23]. 

In our IPD review, an immediate repair strategy, i.e., within one day, thus including the on-table repair, emerged as a significant risk factor for treatment failure at the multivariable analysis, whereas the late approach displayed the lowest percentage of failure, in agreement with the two previously cited metanalysis [145,146].

Indeed, several weeks are usually required for the resolution of the acute inflammatory phase, allowing one to lower the risks associated with extensive reconstructive surgery by reducing inflammation, improving the nutritional status, recovering an adequate immunologic competence, and guaranteeing a correct assessment of the extent of ischemic injury in the case of associated VIs [4,145]. 

Nevertheless, repair success is strictly dependent upon surgical experience and skills [145,149]. Indeed, as emerged in the metanalysis by Wang et al. [145], when exclusively analyzing those patients undergoing repair in a specialized HPB center, a lower rate of repair failure, although not significant, was shown for on-table versus postoperative repair (18.9% vs. 24.7%), apparently contradicting their previous findings.

Therefore, the authors concluded that, since an early repair was more likely attempted by a non-HPB specialist, compared with late repair, nonspecialist attempts could obfuscate the benefits of an on-table repair, whereas more specialist involvement may facilitate the success of late repair [145].

Similar conclusions were also reached in our review, stressing that the repair of the BDI in a non-specialized center represented the most significant risk factor for treatment failure, independently from adopting a surgical or an endoscopic/IR approach.

Therefore, according to the already cited metanalysis [145], to the WSES guidelines [4] and to a recent multicenter study investigating the textbook outcomes of BDIs after cholecystectomy [149], an early referral to an HPB or a tertiary care center should be considered mandatory, aiming at decreasing the likelihood of repair failure, perioperative morbidity, and biliary strictures.

Nevertheless, when the diagnosis is not immediate or logistic constraints limit referral to an HPB/tertiary care center, the “drain now, fix later” strategy with percutaneous drainage of the biloma, targeted antibiotic therapy, and nutritional support seems to be the best approach, rather than attempting a surgical repair [4].

In case of major BDIs, the HJ technique represents the most common and preferred type of surgical reconstruction, being the end-to-end anastomosis potentially associated with increased failure rates, particularly when a concomitant VI is demonstrated [4,149]. 

An anastomotic stricture (AS) represents the most common long-term complication of the above-mentioned reconstructive method, generally occurring after ten to thirty months and in a range between 10 and 20% [150], though minimally invasive (MI) approaches, both laparoscopic and robotic, appear to show promising preliminary results in terms of stricture rate, approximately of 4.5% [151]. 

Main risk factors for stricture are represented by a concomitant VI, a post-repair bile leak and a repair by a non-HPB surgeon, as stated in a recent systematic review and meta-analysis [152]. 

SBC represents another significant long-term complication of BDI repair, with a reported incidence between 2.4 and 10.9%, and potentially requiring a liver transplantation as final treatment [150]. 

In our review, almost one quarter of open HJ developed an AS, in line with the literature [150], whereas no stenosis was reported after MI surgery, though only four cases were included, three robotic and one laparoscopic.

Similarly, SBC was documented in 2.1% of cases, with all patients undergoing subsequently a liver transplantation.

On the other hand, ERCP represents the primary treatment of minor BDIs, with a reported success rate ranging between 87% and 100%, in case of postoperative bile leaks, depending on the grade and location of the fistula [153]. 

Combining, in course of ERCP, biliary sphinterectomy with the placement of a transpapillary stent, both plastic and metallic, aiming to reduce the transpapillary pressure gradient, thus facilitating the bile flow to the duodenum opposite to the site of leak, and represents the preferred approach over a temporary NB drainage, that, although similar in efficacy, might be difficult to bear for the patient [153,154,155]. 

Endoscopic management should be attempted exclusively in those cases with at least a partially documented continuity of the bile tract damage or when the two stumps, proximal and distal, are very close [4,154,155]. 

In the case of non-feasibility or failure of the ERCP, IR procedures, mainly PTBD, can be pursued, though complex, in relation to non-dilated bile ducts, and with success rates ranging between 70% and 80% [156], which are slightly inferior to endoscopic techniques.

Moreover, PTBD allows one to perform an extraluminal percutaneous endoscopic rendez-vous procedure with stent placement in order to restore the continuity of the bile duct [157,158]. 

Lastly, ERCP represents the first-line treatment of benign biliary strictures after cholecystectomy, with the multi-stenting approach considered to be the most effective method, although impaired by a recurrence rate up to 30% within 2 years from stent removal [159]. 

The major advancements currently reported in the endoscopic and IR management of BDIs and the associated improved outcomes [160,161] might explain the results emerged at the sensitivity analysis of the non-surgical group, showing a no longer existing correlation between the severity of the damage pattern and the treatment failure over the latest years.

Even if we have taken a thorough and systematic approach to individual predictive factors using all obtainable IPD collected in published studies until December 2023, making this review a unique contribution to the global evidence base for the risk assessment of treatment failure in the case of BDI after cholecystectomy, there are several limitations to this study. 

First, since the pooled estimates are based on a retrospective case series and case reports only, the possibility of bias inherent to the original studies, cannot be excluded and might still have influenced the results of this review.

Second, IPD could not be obtained for all studies that the search strategy retrieved, causing to include some missing data in our analysis or to exclude some baseline variables, i.e., emergent and elective cholecystectomies, and despite efforts to contact authors, preventing us to obtain the data from some potentially eligible studies.

Third, predictions were not generated for concomitant multiple BDIs, which represents a limitation of most of the current classifications systems, including that adopted in our study. We indeed decided to assign to each individual case the most serious damage pattern reported, with the risk of missing less severe injuries, that might have influenced the outcome.

## 5. Conclusions

In our IPD review, the management of BDIs after cholecystectomy in a non-specialized center resulted the strongest multivariable predictor of treatment failure, followed by the severity of the damage pattern recorded. 

Additionally, the failure of the surgical treatment was associated with the timing of repair adopted, with higher chances of failure when an early approach was endorsed, supporting the conclusion of two previous meta-analyses, and adding more exactness to this topic. 

Further prospective studies adopting uniform codification systems and more rigorous definitions, particularly with regard to the time intervals applied, are required to validate and confirm these findings.

## Figures and Tables

**Table 1:** Variables and endpoints analyzed.

Baseline Variables	Primary Composite Endpoint
Demographics Technique of cholecystectomy (open vs. laparoscopic) Conversion to open and cause Grade of BDI Associated VI and pattern Timing of BDI diagnosis Timing of surgical repair Methodic of surgical repair Type of endoscopic/IR procedure Site of BDI management (HPB/tertiary care center vs. non-specialized center)	Incidence of treatment intervention failure: Surgical Endoscopic/IR

BDI, bile duct injury; VI, vascular injury; IR, interventional radiology; HPB, hepato-pancreatic-biliary.

**Table 2:** Comparisons of variables by group (upfront surgery vs. upfront endoscopy/IR) [1].

	Upfront Surgery Group (*n* = 224)	Upfront Endoscopy/IR Group (*n* = 109)	*p*-Value
Age, median (IQ range) °	47 (36.2–62)	52 (42–65)	0.065
Male, % (*n*) °	37.1 (75)	42.2 (43)	0.40
Upfront OC, % (*n*)	12.1 (27)	12 (13)	0.90
LC converted to open, % (*n*)	27.9 (55)	10.4 (10)	0.001
LC converted due to BDI, % (*n*)	24.1 (45)	2.3 (2)	<0.0001
Grade of BDI, % (*n*) °°			<0.0001
A	4.9 (11)	27.5 (30)	
B	1.8 (4)	0.9 (1)	
C	5.8 (13)	16.5 (18)	
D	19.6 (44)	9.2 (10)	
E	67.9 (152)	45.9 (50)	
BDI type D and E, % (*n*) °°	87.5 (196)	55.1 (60)	<0.0001
Associated VI, % (*n*)	22.8 (51)	2.8 (3)	<0.0001
Timing of BDI diagnosis, % (*n*) °			<0.0001
within one week	79.5 (151)	43.6 (44)	
after one week	20.5 (39)	56.4 (57)	
Repair HPB/tertiary center, % (*n*)			0.025
yes	79 (177)	89 (97)	
no	21 (47)	11 (12)	
Failure rate, % (*n*)	41.1 (92)	20.2 (22)	<0.0001
Mortality rate, % (*n*)	10 (4.5)	0 (0)	0.025

° Data were not reported in some cases. °° According to the Strasberg’s classification. IR, interventional radiology; IQ, inter-quartile; OC, open cholecystectomy; LC, laparoscopic cholecystectomy; BDI, bile duct injury; VI, vascular injury; HPB, hepato-pancreatic-biliary.

**Table 3:** Comparisons of variables by group (surgical failure vs. surgical non-failure) [1].

	Surgical Failure Group (*n* = 92)	Surgical Non-Failure Group (*n* = 132)	*p*-Value
Age > 70 years, % (*n*) °	12.2 (9)	13.6 (15)	0.95
Male, % (*n*) °	41.5 (34)	34.2 (41)	0.37
Upfront OC, % (*n*)	18.4 (17)	7.6 (10)	0.024
LC converted to open, % (*n*)	50.7 (38)	13.9 (17)	0.001
LC converted due to BDI, % (*n*)	45.3 (34)	9 (11)	0.001
BDI type D and E, % (*n*) °°	94.6 (87)	82.6 (109)	0.014
Associated VI, % (*n*)	26 (24)	20.4 (27)	0.40
Timing of BDI diagnosis, % (*n*) °			0.57
within one week	82.7 (62)	77.4 (89)	
after one week	17.3 (13)	22.6 (26)	
Timing of surgical repair, % (*n*) °			0.001
Immediate (within 1 day)	59.3 (48)	34.1 (42)	
Early (>1 day < 1 week)	11.1 (9)	24.4 (30)	
Delayed (>1 week < 6 weeks)	21 (17)	21.2 (26)	
Late (>6 weeks)	8.6 (7)	20.3 (25)	
Repair HPB/tertiary center, % (*n*)			<0.0001
yes	60.9 (56)	91.7 (121)	
no	39.1 (36)	8.3 (11)	

° Data were not reported in some cases. °° According to the Strasberg’s classification. OC, open cholecystectomy; LC, laparoscopic cholecystectomy; BDI, bile duct injury; VI, vascular injury; HPB, hepato-pancreatic-biliary.

**Table 4:** Multivariable analysis results.

Preoperative Risk Factors Contributing to Surgical Failure Variable	*p*-Value	Odds Ratio (95% CI)
Injury type D and E °	0.0094	4.214 (1.423 to 12.48)
Immediate attempt of surgical repair	0.0113	2.219 (1.197 to 4.114)
Repair in a non-specialized center	0.001	4.169 (1.783 to 9.745)

° According to the Strasberg’s classification.

**Table 5:** Comparisons of variables by group (endoscopy/IR failure vs. endoscopy/IR non-failure) [1].

	Endoscopy/IR Failure Group (*n* = 22)	Endoscopy/IR Non-Failure Group (*n* = 87)	*p*-Value
Age > 70 years, % (*n*) °	11.1 (2)	16.9 (14)	0.80
Male, % (*n*) °	44.4 (8)	41.7 (35)	0.96
Upfront OC, % (*n*)	19 (4)	11.1 (9)	0.33
LC converted to open, % (*n*)	11.1 (2)	10.3 (8)	0.92
LC converted due to BDI, % (*n*)	5.8 (1)	1.4 (1)	0.55
BDI type D and E, % (*n*) °°	81.8 (18)	48.3 (42)	0.001
Associated VI, % (*n*)	13.6 (3)	0 (0)	0.006
Timing of BDI diagnosis, % (*n*) °			0.48
Within one week	53.3 (8)	41.9 (36)	
After one week	46.7 (7)	58.1 (50)	
Endoscopic treatment, % (*n*)	81.8 (18)	86.2 (75)	0.85
IR treatment, % (*n*)	68.2 (15)	43.7 (38)	0.07
Repair HPB/tertiary center, % (*n*)			<0.0001
yes	59.1 (13)	96.6 (84)	
no	40.9 (9)	3.4 (3)	

° Data were not reported in some cases. °° According to the Strasberg’s classification. IR, interventional radiology; OC, open cholecystectomy; LC, laparoscopic cholecystectomy; BDI, bile duct injury; VI, vascular injury; HPB, hepato-pancreatic-biliary.

**Table 6:** Multivariable analysis results.

Preoperative Risk Factors Contributing to Endoscopy/IR Failure Variable	*p*-Value	Odds Ratio (95% CI)
Injury type D and E °	0.02	4.607 (1.263 to 16.804)
Repair in a non-specialized center	0.0002	18.620 (4.093 to 84.710)

° According to the Strasberg’s classification. IR, Interventional radiology.

## Data Availability

The raw data supporting the conclusions of this article will be made available by the authors on request.

## Appendix A

**MEDLINE**

(((((Laparoscop* OR Celioscop* OR Coelioscop* OR Abdominoscop* OR Peritoneoscop* OR lap) AND (cholecystectom* OR colecystecto* OR chole) OR “Cholecystectomy, Laparoscopic” [Mesh]) AND ((((injur* OR bile leak* OR biliary leak* OR transection* OR occlusion* OR stricture* OR stenosis* OR obstruct* OR laceration* OR damage*) OR (harm* OR convers* OR “Peritonitis” [MeSH] OR peritonitis OR “Cholestasis” [Mesh] OR Cholestas*) OR ((biliary OR bile) AND stas*)) AND (bile duct* OR biliary tract*)) OR “Biliary Tract/injuries” [Mesh])) AND (English [LA]))) NOT (animals [MeSH] NOT (humans [MeSH] AND animals [MeSH])).

**SCOPUS**
*All fields*

(Laparoscop* OR Celioscop* OR Coelioscop* OR Abdominoscop* OR Peritoneoscop* OR lap) AND (cholecystectom* OR colecystecto* OR chole) AND (injur* OR bile leak* OR biliary leak* OR transection* OR occlusion* OR stricture* OR stenosis* OR obstruct* OR laceration* OR damage*) OR (harm* OR convers* OR peritonitis OR Cholestas*) OR (biliary OR bile AND stas*) AND (bile duct* OR biliary tract*) AND (English) AND NOT (animals) AND NOT (humans AND animals).

**Table A1:** Basic characteristics of the included studies.

Author	Publication Year	Country	N Cases	M/F	Age (y) °	Initial Classification Used	Primary Repair Site (HPB-H/C-H)
Parmeggiani et al. [137]	2010	Italy	3	1/2	52 (45–56)	none	1/2
Bernhardt et al. [120]	2010	Austria	1	0/1	49	none	1/0
Chiruvella et al. [121]	2010	USA	1	0/1	65	Bismuth	1/0
Moo-Young et al. [102]	2010	USA	1	0/1	64	none	1/0
Humes et al. [107]	2010	England	3	2/1	42 (35–63)	none	3/0
Marin et al. [110]	2010	USA	1	1/0	68	none	0/1
Selvaggi et al. [60]	2010	Italy	1	0/1	40	none	0/1
Ganguly et al. [40]	2010	USA	1	1/0	51	none	1/0
Schmidt et al. [32]	2010	Germany	14	n.r.	n.r.	Neuhaus	14/0
Hwang J.C. et al. [37]	2011	Republic of Korea	1	0/1	31	none	1/0
Ball et al. [125]	2011	USA	1	0/1	27	none	1/0
Brunet et al. [103]	2011	Canada	1	0/1	48	none	1/0
Palermo et al. [94]	2011	USA	1	0/1	43	none	0/1
Lau et al. [119]	2011	USA	1	1/0	30	none	1/0
Mazer et al. [91]	2011	USA	7	1/6	41 (22–71)	none	7/0
Yan et al. [73]	2011	China	1	1/0	41	Strasberg	1/0
Maurea et al. [93]	2011	Italy	1	0/1	41	Bismuth	1/0
Hwang S. et al. [58]	2011	Republic of Korea	1	0/1	48	none	1/0
Romano O. et al. [51]	2011	Italy	2	1/1	50 (45–55)	none	0/2
Choi et al. [39]	2011	Republic of Korea	1	0/1	34	none	1/0
Donatelli et al. [126]	2012	France	1	1/0	51	none	1/0
Strasberg et al. [118]	2012	USA	8	0/8	n.r.	Strasberg	8/0
Truant et al. [75]	2012	France	3	2/1	35 (25–43)	Strasberg	1/2
Mohamadnejad et al. [109]	2012	Iran	1	0/1	68	none	1/0
Sasahira et al. [76]	2012	Japan	1	0/1	58	none	0/1
Olmez et al. [77]	2012	Turkey	1	1/0	53	Strasberg	1/0
Addeo et al. [101]	2013	France	6	2/4	39 (28–46)	Strasberg	0/6
De Werra et al. [122]	2013	Italy	9	n.r.	n.r.	Strasberg	9/0
Denjalic et al. [96]	2013	Bosnia	1	1/0	43	none	1/0
Wojcicki et al. [79]	2013	Poland	2	0/2	18 (15–21)	Strasberg	2/0
Mungai et al. [92]	2013	Italy	1	0/1	42	none	1/0
Macedo et al. [66]	2013	USA	1	0/1	58	none	1/0
Majumder et al. [59]	2013	USA	1	0/1	58	none	1/0
Parlak et al. [61]	2013	Turkey	7	1/6	44 (30–82)	none	7/0
Paik et al. [28]	2013	Republic of Korea	1	1/0	76	none	1/0
Gluszek et al. [138]	2014	Poland	14	3/11	45 (29–84)	ATOM	13/1
Buturovic et al. [106]	2014	Bosnia	1	1/0	40	none	1/0
Crema et al. [116]	2014	Brazil	1	1/0	41	Bismuth	1/0
Odemis et al. [105]	2014	Turkey	1	0/1	45	Stewart-Way	1/0
Donatelli et al. [98]	2014	France	16	6/10	55.5 (31–89)	Strasberg	16/0
Shokouh-Amiri et al. [111]	2014	USA	1	0/1	71	none	1/0
Jadrijevic et al. [80]	2014	Croatia	1	0/1	36	Bismuth	0/1
Judd et al. [104]	2014	USA	1	1/0	68	none	1/0
Laux et al. [83]	2014	USA	1	0/1	48	none	1/0
Jalali et al. [97]	2014	USA	1	0/1	45	Strasberg	0/1
Yaqub et al. [52]	2014	Norway	1	1/0	32	Strasberg	1/0
Sofi et al. [33]	2014	USA	3	2/1	46 (27–61)	none	3/0
Anantha et al. [34]	2014	USA	2	1/1	39.5 (29–52)	none	2/0
Sugawara et al. [29]	2014	Japan	14	11/3	51 (27–81)	Strasberg	14/0
Artifon et al. [141]	2015	Brazil	1	0/1	63	none	1/0
Velidedeoglu et al. [81]	2015	Turkey	12	2/10	n.r.	Strasberg	12/0
Odemis et al. [142]	2015	Turkey	1	1/0	53	none	1/0
Prasad et al. [67]	2015	India	1	0/1	36	Strasberg	1/0
Rai et al. [57]	2015	USA	1	0/1	54	none	1/0
Pekolj et al. [30]	2015	Argentina	15	7/8	44 (23–65)	Strasberg	5/10
Curcio et al. [124]	2016	Italy	1	0/1	36	none	1/0
Merrick et al. [85]	2016	England	1	0/1	48	Bismuth	0/1
Takahashi et al. [49]	2016	Japan	2	0/2	78 (73–83)	none	2/0
Hoepfner et al. [38]	2016	USA	1	1/0	79	none	1/0
Sasaki et al. [131]	2017	Japan	1	1/0	78	none	1/0
Dokmak et al. [88]	2017	France	3	1/2	38 (26–45)	none	3/0
Dokmak et al. [87]	2017	France	1	0/1	59	none	1/0
Ko et al. [108]	2017	Republic of Korea	1	1/0	51	none	1/0
Zoričić et al. [86]	2017	Croatia	1	1/0	78	Strasberg	1/0
Park et al. [70]	2017	Republic of Korea	1	1/0	67	none	1/0
Xu et al. [140]	2017	USA	1	0/1	32	none	1/0
Odemis et al. [45]	2017	Turkey	1	1/0	58	none	1/0
Meek et al. [53]	2018	USA	1	0/1	44	none	1/0
Kohn et al. [132]	2018	USA	7	n.r.	n.r.	Strasberg	7/0
Dousse et al. [112]	2018	France	1	0/1	55	Bismuth	0/1
Elmunzer et al. [127]	2018	USA	1	0/1	43	none	1/0
Rifatbegovic et al. [41]	2018	Bosnia	1	0/1	21	Strasberg	1/0
Runge et al. [65]	2018	USA	1	0/1	24	none	1/0
Lim et al. [54]	2018	Australia	1	0/1	44	none	0/1
Kotecha et al. [113]	2019	Australia	1	0/1	56	none	1/0
Nezami et al. [136]	2019	USA	7	5/2	60 (44–76)	none	7/0
Machado et al. [134]	2019	Brazil	1	0/1	24	none	1/0
Bonati et al. [133]	2019	Italy	1	0/1	45	none	1/0
Acquafresca et al. [129]	2019	Argentina	1	1/0	64	none	1/0
Ayloo et al. [128]	2019	USA	1	0/1	36	Strasberg	1/0
Battal et al. [100]	2019	Turkey	13	4/9	46 (19–65)	Strasberg	13/0
Desai et al. [123]	2019	India	1	0/1	35	none	1/0
Kumar et al. [82]	2019	India	1	0/1	42	Strasberg	1/0
Kwak et al. [71]	2019	Republic of Korea	8	2/6	49 (29–77)	Bismuth	8/0
Özdemir et al. [63]	2019	France	1	0/1	42	Strasberg	0/1
Kohga et al. [56]	2019	Japan	2	1/1	82.5 (81–84)	none	0/2
Gallagher et al. [36]	2019	USA	1	0/1	67	Strasberg	1/0
Martines et al. [27]	2019	Italy	43	19/24	50 (15–81)	Strasberg	34/9
Lubikowski et al. [130]	2019	Poland	1	0/1	26	Bismuth	1/0
Drs et al. [117]	2020	Czech Republic	1	1/0	45	Stewart-Way	1/0
Tsai et al. [95]	2020	Taiwan	2	1/1	55.5 (53–58)	Strasberg	1/1
Hite et al. [115]	2020	USA	1	0/1	31	none	0/1
Fan et al. [62]	2020	China	1	1/0	65	Stewart-Way	0/1
Vasilidias et al. [64]	2020	Greece	1	0/1	46	Strasberg	0/1
Sondhi et al. [47]	2020	USA	1	1/0	87	Bergman	1/0
Ferrada et al. [31]	2020	Chile	1	1/0	65	Strasberg	1/0
Bowen et al. [99]	2021	Ireland	1	0/1	30	none	1/0
Romano L. et al. [69]	2021	Italy	1	0/1	18	none	1/0
Wei et al. [90]	2021	USA	1	1/0	85	Strasberg	0/1
Lin et al. [135]	2021	Taiwan	1	1/0	50	none	1/0
Mistry et al. [26]	2021	India	2	0/2	53 (30–76)	Strasberg	2/0
Anwar et al. [84]	2022	Indonesia	1	0/1	32	none	1/0
Mayer et al. [114]	2022	France	1	0/1	70	none	1/0
Torretta et al. [68]	2022	Italy	1	1/0	65	Strasberg	1/0
Garcia et al. [74]	2022	Brazil	1	0/1	39	none	1/0
Sharma et al. [72]	2022	USA	1	0/1	62	none	1/0
Kakati et al. [42]	2022	Lebanon	4	2/2	38 (30–50)	none	4/0
Emara et al. [46]	2022	Egypt	1	1/0	58	none	1/0
D’Ovidio et al. [35]	2022	Italy	1	1/0	60	Hannover	0/1
Robles-Medrandra et al. [25]	2022	Ecuador	1	0/1	24	Strasberg	1/0
Sucandy et al. [89]	2023	USA	1	0/1	43	Strasberg	1/0
Swallow et al. [139]	2023	Tanzania	2	0/2	43 (40–46)	Bismuth	2/0
Hristov et al. [55]	2023	Bulgaria	1	0/1	33	Strasberg	1/0
Rott et al. [48]	2023	Germany	1	1/0	68	none	1/0
Abughararah et al. [43]	2023	Saudi Arabia	1	1/0	85	Strasberg	1/0
Rathore et al. [78]	2023	India	1	0/1	43	Bismuth	0/1
Selleslag et al. [50]	2023	Belgium	1	0/1	74	none	1/0
Marti Romero et al. [44]	2023	Spain	1	0/1	79	Strasberg	0/1

° If more than one case, age was expressed in median with range. HPB-H, hepato-pancreato-biliary hospital; C-H, community hospital; n.r., not reported.

**Table A2:** Our series of patients with BDI.

Pt n.	Age (y)/ Sex	Indication for Surgery	Primary Surgery	BDI Type °	VI	Timing Diagnosis	Initial Treatment/ Site	Timing Surgical Repair	Further Treatment	Outcome
1	78/M	acute calculous cholecystitis	LC, converted	E2	-	4 d	HJ/ HPB-H	9 d	-	death
2	42/F	lithiasis, elective	OC	E2	-	io	HJ/ HPB-H	14 d	PTBD, re-do HJ for AS	no further complications
3	50/F	lithiasis, elective	LC	D	-	5 d	ERCP + stenting/ HPB-H	-	ERCP + ballooning for stricture	no further complications
4	68/F	lithiasis, elective	LC	E2	-	58 d	ERCP + stenting, PTBD/HPB-H	-	-	no further complications
5	40/F	lithiasis, elective	OC	D	-	4 d	E-E A/ C-H	5 d	PTBD, HJ for AS	no further complications
6	22/M	acute calculous cholecystitis	LC	E2	-	26 d	HJ/ HPB-H	29 d	re-do HJ + LH for AS	no further complications
7	67/M	lithiasis, elective	LC, converted	E2	-	45 d	HJ/ HPB-H	49 d	-	no further complications
8	79/M	acute calculous cholecystitis	LC, converted	D	-	io	HJ/ HPB-H	1 d	-	no further complications
9	84/M	acute calculous cholecystitis	LC	E4	-	3 d	ERCP + stenting/ C-H	9 d	HJ	no further complications
10	49/M	acute acalculous cholecystitis	LC	E4	-	11 d	RH + HJ/ HPB-H	81 d	-	no further complications
11	62/M	lithiasis, elective	LC	E2	-	148 d	HJ/ HPB-H	153 d	-	no further complications
12	67/M	lithiasis, elective	LC	E3	-	10 d	ERCP + stenting/ C-H	24 d	HJ	no further complications
13	34/F	lithiasis, elective	LC	C	-	5 d	clips placement/C-H	7 d	ERCP + stenting/ E-lap	no further complications
14	32/F	lithiasis, elective	LC	E4	-	io	T-tube insertion/C-H	i.o.	HJ	no further complications
15	33/F	lithiasis, elective	LC	E2	-	3 d	HJ/ HPB-H	4 d	-	no further complications
16	87/M	lithiasis, elective	LC	E4	RHA	io	HJ + VR/ HPB-H	1 d	-	no further complications
17	36/M	acute calculous cholecystitis	LC	E2	-	4 d	HJ/ C-H	12 d	PTBD/ ERCP + stenting for stricture	recurrent cholangitis
18	49/M	acute calculous cholecystitis	LC converted	D	-	io	primary suture repair/C-H	i.o.	PTBD/ robotic HJ	no further complications
19	73/F	lithiasis, elective	LC	C	-	16 d	ERCP + stenting/ C-H	36 d	laparoscopic leak repair	no further complications

° According to the Strasberg’s classification. BDI, bile duct injury; VI, vascular injury; LC, laparoscopic cholecystectomy; HJ, hepatico-jejunostomy; HPB-H, hepato-pancreato-biliary hospital; OC, open cholecystectomy; io, intraoperative; PTBD, percutaneous transhepatic biliary drainage; AS, anastomotic stricture; ERCP, endoscopic retrograde colangio-pancreatography; E-E A, end-to-end anastomosis; C-H, community hospital; LH, left hepatectomy; RH, right hepatectomy; E-lap, exploratory laparoscopy; RHA, right hepatic artery; VR, vascular reconstruction.

**Table A3:** Sensitivity analysis: the pooled OR was reassessed after excluding the studies published before 2013.

**1. Upfront surgery group**		
**Preoperative risk factors contributing to Surgical Failure** **Variable**	***p*** **-value**	**Odds ratio** **(95% CI)**
Injury type D and E °	0.028	3.845 (1.153 to 12.82)
Immediate attempt of surgical repair	0.0015	3.373 (1.594 to 7.14)
Repair in a non-specialized center	0.0001	8.317 (2.878 to 24.035)
**2. Upfront endoscopy/IR upfront group**		
**Preoperative risk factors contributing to rndoscopy/IR gailure** **Variable**	***p*** **-value**	**Odds ratio** **(95% CI)**
Repair in a non-specialized center	<0.0001	34.2 (6.45 to 181.32)

° According to the Strasberg’s classification. IR, interventional radiology.
